# Children’s agency in parent–child, teacher–pupil and peer relationship contexts

**DOI:** 10.1080/17482631.2019.1565239

**Published:** 2019-02-01

**Authors:** Sevtap Gurdal, Emma Sorbring

**Affiliations:** Centre for Child and Youth Studies, Department for Social and Behavioural Studies, University West, Trollhättan, Sweden

**Keywords:** Agency, peer relationships, parent relationship, teacher relationship, well-being

## Abstract

**Purpose**: The aim of this study was to examine children’s perception of their agency in different relationship contexts. Historically, most studies conducted in Sweden concerning children’s agency, in relation to their self-efficacy and perceptions of their effectiveness as agents, have been carried out in school situations or other institutional organizations. Past research has shown that children’sagency has positive links to health, school achievement and/or adjustment. **Method:** Interviews were conducted with 103 10-year-old Swedish children to examine three relationship contexts: parent–child, teacher–pupil, and peer relations. Vignettes about the different contexts were presented to the children and their answers were analysed with thematic analysis. **Results:** The results show that children think of their agency differently depending upon which relationship context they find themselves in. Most perceived agency are found insituations with peers, and children perceive they have the least agency with teachers. In situations with parents, children think they would react with more resistance than with peers and teachers. It is mainly with other children that they would show assertiveness and try to find asolution together, while they would be more emotional and perceive less power with adults. **Conclusion:** We conclude that children make adistinction in their perception of agency depending upon the relationship context. These findings can be relevant for helping children receive more agency in all contexts, which might have apositive impact on health and adjustment.

## Introduction

There are different ways to achieve a healthy life and a sense of well-being. Greenaway et al. (2015) show that health can be affected by a number of variables, for example perception of personal control. Perceived control is one part of personal agency, along with positive self-esteem, self-efficacy and purpose in life (Côté, 1997). In this paper the term child agency will refer to children’s beliefs that they can affect an outcome; to have self-efficacy and be effective as agents. We argue that agency beliefs vary depending on the specific situation or relationship that is the target of their influence attempts. This means that earlier experiences that children have may affect the way they choose to act in a new, but similar, situation (Kuczynski & De Mol, 2015). That is, children learn from past experiences how effective they are when they choose to act and these lessons affect their sense of their own efficacy in particular situations. Bandura (2001) defines this as personal self-efficacy which is defined as knowing and predicting what kind of outcome a certain action will have or behaving intentionally with the expectation of achieving a specific outcome.

Historically, children have not been studied as agencies, instead they have for example been perceived as passive “human becomings” (Matthews, 2007). However, renewed interest in children’s agency, in both research and in various contexts, has led to perceptions of the child as an agent with an active role in development (James & James, 2004; Kuczynski & De Mol, 2015). Defining children as agentic is to grant them a mind of their own and with their own will and thereby acknowledge their self-efficacy and personal control. Children are active in creating meaning in life; they are not only recipients but also creators (Kuczynski, 2003). Moreover, they possess the capacity to engage in society and are capable of participating in decision making (Mayall, 1994).

Historically, most studies conducted in Sweden concerning children’s agency have been done in school settings with a focus on the teacher–pupil dyad. Little is known about children’s agency in other contexts or how the child’s agency in different relationship contexts either differs or is correlated. We propose that a person’s belief in their own capacity to exert control over outcomes depends on context, and that levels of expressed agency can vary within the same person depending on context. In addition, it might then be possible to assist children so they can feel more agentic in a variation of contexts. The goal of the current study is to contribute knowledge about children’s perception of their own agency by examining how 10-year-old children in Sweden think of their agency in different relationship contexts. Three relationship contexts were examined: parent–child relations, teacher–pupil relations and peer relations. All three contexts are situations that most children experience in everyday life when exposed to interaction with adults and other children.

Children’s perception of agency is related to their experience of the efficacy of their actions. Children’s perception of agency has been studied especially in older children and their peer relationships. Researchers have found that children who describe themselves as more agentic, measured as levels of action, planning of action and action effectiveness, also report less trouble in school, for example less impulsiveness and more awareness of why they do certain thing and not only how they do it (Vallacher & Wegner, 1989). Agency is also positively related to health and school adjustment and shows. Children’s belief in their own self-efficacy has a positive influence on health, well-being and adjustment (externalizing and internalizing behaviour) as well as school grades (Daniels et al., 2014; Dignath, Buettner, & Langfeldt, 2008; Greenaway et al., 2015; Grob, Little, Wanner, Wearing, & EURONET, 1996; Gurdal, Lansford, & Sorbring, 2015; Juang & Silbereisen, 2002; Lopez & Little, 1996; Musher-Eizenman, Nesselroade, & Schmitz, 2002; Stetsenko, Little, Oettingen, & Baltes, 1995; Walls & Little, 2005).

Different factors can have an influence on children’s experience and practice of agency and each child, regardless of socioeconomic status, is influenced by different relational contexts they encounter. Each context has different expectations and power dynamics, and affords different possibilities for action. According to Kuczynski and De Mol (2015) an individual has access to three resources; individual-, relational- and cultural resources, which contribute to variations in children’s experience and effectiveness as agents in different situations. For example, children vary in *individual resources* such as cognitive abilities, physical strength, and possessions because of differences in experiences, development and genetic endowment. These differences in capacities may affect how successful children are in, for example, persuasive negotiations with their parents. Children also vary in *relational resourc*es such the number and quality of close relationships they can draw on to support their goals and actions. In this regard, for most children, having a parent, teacher or other person who is responsive to their requests and communications is a particularly important source of successful acts of agency. Children also vary in *cultural resources* that support their agency, including norms for acceptable power relations between children and adults, and expectations about obedience and respect for authority. Cultural norms with regard to child and adult relationships have undergone great changes during the last century. For example, today children in contemporary Western societies have more of an opportunity to offer their opinion and tell adults what it is like to be a child (Matthews, 2007). This change was documented about 25 years ago in a Swedish study where most parents in Sweden reported viewing themselves and their children as equals who can discuss various issues with each other (Halldén, 1991).

All relationships are important in different ways (Laursen & Bukowski, 1997); some relationships, like the parent–child relationship, are long-term, and some, like peer relationships, are more temporary. According to Laursen and Bukowski (1997) there are three things that can influence the relationship. The first is the permanence, that is, if the relationship is voluntary or obligatory. The second is what kind of power relationship the individuals have between each other, that is if it is hierarchical and egalitarian or not. Gender is the third factor that they point out, whether it is a same-sex or cross-sex relation. In this study three relationship contexts are examined: children’s relationships with their parents, teachers and peers. All can be classified as relationships since they are repeated and have a meaning (Laursen & Bukowski, 1997).

The family is the single most frequent relationship context in which children, especially those of a young age, spend time. Considered as a context for children’s agency, the parent–child relationship may offer children more leeway for expressing agency than other adult–child contexts. Although the parent–child relationship is unequal in power, Kuczynski (2003) argues that this power difference should be considered a dynamic, interdependent asymmetry where parents may be more receptive or vulnerable to children’s influence than non-parental or non-intimate adult–child relationships such as with teachers or unfamiliar adults. Research with Swedish families suggest it is the norm for parents and children to expect parental receptivity to child influence. That is, children can be active agents just like adults (Harach & Kuczynski, 2005). A Swedish study revealed that parents in Sweden did not display obvious authority in their parenting; instead parents and children reported that parents and children made decisions in consultation with each other (Björnberg, 2002). More recent research found that 72% of Swedish adolescents described their families as democratic and that it was possible to influence their parents in conflict situations (Persson, Stattin, & Kerr, 2004). Similarly, Sorbring (2005) found that younger school age children wanted to have influence in conflict situations with their parents. Sorbing found that children expressed their agency in three different ways in conflicts with parents: (1) actively confronting the parent by, for example, using different kinds of reasoning and arguments; (2) goal-oriented behaviour, such as doing something positive to get the parent to show goodwill; and (3) choosing not to confront the parent, such as going to their room when conflict begins or standing quietly until the parent has calmed down. All three strategies are in some way deliberate, and reveal how children use their agency to act to get a certain reaction or outcome. Furthermore, research reveals that adolescents’ participation in family discussions also increases children’s belief in higher personal agency (Jutengren, 2004).

Another relational context in which children spend time almost every day is the institutional context of the school, with their teachers. As with parents, children also use different strategies to influence teachers and other adults (Markström & Hallden, 2009). A child can, for example, negotiate and try to take control of a situation, or a child can choose to be silent and avoid conflict if he/she does not agree with the teacher. In Sweden, it is expected that children are treated with respect and should be taught about their rights (Harcourt & Hägglund, 2013). School is a common place to teach children about their rights and how to practise them. According to the Swedish curriculum, teachers are supposed to encourage children’s agency by, for example, letting them take responsibility and be involved in decisions about their lives (Lgr11, 2011). This is related to the goal of teaching children more about how to become a citizen and about democratic values in society (Harcourt & Hägglund, 2013). Some of the UNCRC declarations can even be found in the Swedish curriculum, including, for example, democratic values and the requirement of putting the child’s best interests first. The majority of schools in Sweden have class or student councils as part of the institutional organization (Skolverket, 2001), thus giving children an opportunity to make their voices heard.

A third relational context for children’s expression and experience of agency, studied in this paper, is the peer relationship. Studies of peer relations have shown that children interpret signals they receive from adults and then reproduce the situation in future behaviour and in peer relationships (Laursen & Bukowski, 1997). Peer relationships are considered as not obligatory and often based on equality. Although peer relationships can sometimes be hierarchal in power, more often, peers have a horizontal relationship where egalitarianism is the norm (Laursen & Bukowski, 1997). This variation in power structure is shown in one study of Swedish preschool children and revealed that the power relations in a play situation can vary due to age. Age was an important factor for determining who could be part of the game or which child made the decisions in a specific situation; the older peers had more influence than younger peers (Löfdahl & Hägglund, 2006).

Although there are contextual differences in the relationships, or as Laursen and Bokowski put it, “relationships change as environments change” (1997, p. 748), there are also qualities that repeat from one context to another. A horizontal child–parent relationship, that is a relationship with equality between the individuals, can for example be reused with peers (Russell, Petitt & Mize, 1998).

### The present study

Research on children’s experience and practice of agency has been conducted in single contexts, including parent–child, teacher–pupil, and peer relationships. However, there is a theoretical basis to expect that children’s experiences and perception of themselves as effective agents may vary depending on specific qualities of different relational contexts. The purpose of this study is to explore children’s perceptions of their agency across all three contexts. Three research questions are posed:

1. How do children perceive that people in different contexts react in situations where the child shows agency by resisting demands or initiating demands?
2. How do children believe they, themselves, will react if their acts of agency are not respected?
3. Do children, as a group, perceive that their experience of agency varies depending upon whether they are interacting with children or adults? Do their perceptions of their agency vary if the adult is a parent or a teacher?

## Method

### Participants

Study participants were 10-year-old children (N = 103, 50 girls and 53 boys) recruited from six different schools in western Sweden from a socioeconomically diverse population. The children were on average 9.84 years old (SD = 0.395). The family environment of the participants was as follows: 55.1% of their parents were married, 17.8% were cohabiting and 14% were legally divorced or were living apart. The remaining 13.1% of children lived in single parent families and did not have both parents involved in their lives. Eighty-six per cent of the children had at least one sibling. Mothers were on average 40.31 years old (SD = 4.86) and fathers 42.91 (SD = 5.50). The average level of education was 13.34 years (SD = 4.17) for mothers and 13.22 (SD = 3.92) for fathers.

### Procedure

The heads of six schools were contacted. After obtaining their approval, 182 recruitment letters were sent to the parents of the children in grade three. Of these 182 families, 103 parents gave permission for their child to be interviewed and the children were contacted to obtain their assent. Interviews with children were conducted by one of two researchers in school or after school either at after-school centres or at the child’s home. Every interview took place in private, out of earshot of others. A modest gift was given to each child and the school. Both parents and children were informed about confidentiality and the right to terminate the interviews at any time. All procedures, research design and measures were sent and reviewed by the regional ethical committee (Dnr 1011-13). Parents provided written consent and youth provided assent.

### Interviews

Three vignettes constructed by the researchers were presented to the children during the interviews. Each vignette represented a different relationship context: parent–child, teacher–pupil, and peer–peer. The vignettes were chosen to reflect common situations from their daily lives that children could easily recognize as attempts to exercise their agency. Children were asked: (1) what would happen if they acted by refusing or resisting a request from the other person in the relationship; and (2) how he or she would react if agency were blocked or refused by the other person.

In the parent–child context, children were asked:

Imagine yourself and your mother/father in a hurry one morning. You must leave home as soon as possible. You refuse to leave. What would happen if you told your parent that you think it is hard for you when there is a lot of stress in the morning? What would you do if your mother/father lifted you up and carried you to the car?

In the teacher–pupil context children were asked to imagine the following situation:

It is raining cats and dogs outside and you are supposed to have a break in school. During the break earlier that day you were outside so most of the children’s clothes are still wet. You do not want to go out, but your teacher says you must. You refuse to go out. What would happen if you said that you want to vote about going out or not? What would you do if your teacher said that you must go out and that’s final?

In the peer–peer context children were asked:

You and one of your friends are about to play, but you want to play different games. Your friend will not accept your game suggestion and you refuse to give up. What would happen if you told your friend that it was a very long time since you played your game and that you really want to play it? What would you do if your friend said that he/she has played your game a lot and is tired of playing it?

The strategy was to have children imagine themselves expressing agency by asserting their preference, and to get their reactions to someone blocking their self-selected choices. We used open-ended questions since we thought that it would be difficult to explain agency to 10-year-old children but also wanted to avoid giving agency a set value.

### Analysis

All children’s answers were written down by the interviewer and were not compiled until all 103 interviews were finished. We did not use any audio recording devices because the vignettes were a complement to a larger battery of questionnaires. The interviews were analysed by two researchers using thematic analysis. The purpose of thematic analysis is to identify, analyse, and report patterns (themes) within data (Braun & Clarke, 2006, p. 6). Braun and Clarke (2006) outline six phases of the thematic analysis procedures: becoming familiar with the data; generating initial codes; searching for themes; reviewing themes; defining and naming themes; and producing the report. These steps were followed in the analyses: two researchers began by reading through all the data and keywords, and initial codes that were related to the research questions were marked. Another reading was completed, and the keywords were categorized into themes; these are presented in the result section, Tables I and II. The data were at first gender coded; however, no gender differences were found. Therefore, all data were analysed together, not divided by gender of the child or the parent in the vignettes.

**Table I.** Children’s perceptions of responses to their agency in three relational contexts.

	Parent context	Teacher context	Peer context
**Child agency suppressed**			
Child agency ignored	X	X	–
Child agency reprimanded	–	X	–
**Child agency supported**			
Child agency acknowledged but not rewarded	X	X	X
Child agency taken into account	X	X	X
Child agency rewarded	X	–	X

*Note* X = the parent, teacher or peer context has at least one citation in that subtheme

## Results

The analyses focused on Swedish children’s perceived agency across three relationship contexts. The results were divided into two sections. The first reports children’s perceptions of how the other person in the relationship (parents, teacher, peer) would react in situations where children express their agency by asserting their preferences. The second section presents analyses of children’s perceptions of their own reactions to having their acts of agency refused or blocked. Each theme with subthemes is presented in Table I.

### Children’s perceptions of the other person in different relationship contexts

When showing agency by stating their preferences or pursuing their own goals, children perceived reactions of others differently depending on the social context. Two main themes, *suppressed agency* and *supported agency*, were used to describe children’s descriptions of how their parent, teacher or peer would react if they attempted to acted with agency.

#### Theme 1: suppressed agency

Two subthemes were found: *child agency ignored* and *child agency reprimanded*. The first subtheme, child agency ignored, was expressed in the parent and teacher context. This lack of support for agency was evident when children expressed the belief that the adult would not listen at all. Regarding their parents and teachers, the children said, for example: “parents never listen” or “teachers decide for children”. The second subtheme, child agency reprimanded, was solely revealed in interaction with teachers. Children believed that their ideas or opinions would not only be rejected but also would be punished with a reprimand such as a negative evaluation. They thought that the teacher would say “that is a ridiculous idea” or “it is a bad idea”. This belief shows that children not only predicted their attempt would be suppressed but also that they themselves were under-valued.

#### Theme 2: child agency supported

Some children predicted that their attempt to express agency would be encouraged by their parent, teacher or peer and described their agency as supported, which is the second main theme in the analyses. The three subthemes in this theme are: *child agency acknowledged but not rewarded, child agency taken into account* and *child agency rewarded*. The first subtheme, when the child’s agency is acknowledged but refused, is a situation when the child is listened to but does not get the effect he or she wants. This theme is found in all three contexts. One example is when child predicted a “no” response from his or her teacher, but the teacher also would provide an explanation for the refusal. The teacher could, for example, say that children have to go outside despite the rain because “you have to get fresh air”. Another explanation could be that the child can “put on raingear to avoid getting wet”. These narratives are interpreted as children’s perceptions that their agency would likely be noticed and in some way supported because the teacher explained and responded to the child’s desire. The same explanation scenario is found both with parents and peers. For example, the parents might explain that they understand the child’s feelings: “I understand, but I have to hurry to get there in time” or “I am sorry I have to hurry”.

The second subtheme referred to children’s predictions that their acts of agency would be taken into account. This theme emerged when children reported they believed they would have some influence. For example, the child believed that he or she has not received “no” as an answer, but still has some influence on his or her parents or teacher. Children described the situation as the adults display of understanding and making an effort to accommodate the child’s agency in the situation: “My parent would slow down, or they would become sad and apologize”. Another response that the child thought he or she would get is that the “parent calms down” and says that it is good that the child offers his or her opinion. In some cases, according to the children, the parents would even have solutions for ways to avoid stressful situations in the future, saying something like “we have to set the alarm clock to go off earlier” or “we have to get up earlier”. According to the children, the teacher might say “we can take a vote” or “we can work on democracy now”. This last quote is related to a school theme that the children were working on at the time.

The last subtheme captured children’s perceptions that their act of agency would be rewarded and likely to result in success. This subtheme only occurred in the parent and peer relationship contexts. In the parent relationship context children expressed the feeling of being able to help and take charge. Children gave examples such as “I would try to calm my parent down”, “I could eat my breakfast faster” and “I would put on my clothes myself to help”. Children also said that they could “go to school by myself to save my parents time”. Children explained their acts of agency in a different way in the peer relationship context. This situation revealed a more democratic solution where they could engage in discussion and arrive at a mutual decision. If they and their peers do not want to play the same game, the solutions could be to “play both games”, “make one game out of the two” or to come up with “a new game that both want to play”. This shows that it is not uncommon to have a discussion and come to an agreement between peers. In addition to the democratic solutions, children also thought that they could allow themselves to be more persistent with peers either by complaining or to persuade the friend to do what they want. For example, one child said, “I would say please in a friendly way”. Last, children gave examples showing knowledge that peers can be different from each other: “it depends on the friend” or “I would play with someone else”.

### Children’s beliefs about their agency when their action is not respected

This section reports analyses of children’s predictions of their reactions to negative or uncooperative responses to their acts of agency in parent teacher and peer relationship contexts. The results are compiled under two main themes, *sense of powerlessness* and *child acts with agency*. Each theme with subthemes is presented in Table II.

**Table II.** Subthemes found in each context.

	Parent context	Teacher context	Peer context
**Sense of powerlessness**			
Chooses to surrender	X	X	–
Does not know how to act	X	–	–
**Child acts with agency**			
Emotional resistance	X	–	–
Negotiation and persuasion	–	X	–
Attempts to restore balance of power	X	X	–
Attempts to find a solution	–	–	X

*Note* X = the parent–child, teacher–child or peer context has at least one response in that subtheme

#### Theme 1: sense of powerlessness

The theme *sense of powerlessness* was used to describe children’s reactions when their acts of agency were unsuccessful. Children indicated feelings of being powerless only in the parent–child and teacher–pupil relationship contexts. Two subthemes *chooses to surrender* and *does not know how to act* captured children’s lack of a sense of efficacy when faced with opposition from powerful authorities in their lives. For example, children predicted that they would do nothing or said there is “nothing I can do”, “I have to do as they say”,’ or “I would go out, I do not dare to do anything else, they would call home”. Children also were worried about negative consequences for expressing their views. For example, some children reported that they would be worried that the teacher would call home to tell the parents about the child’s behaviour. These answers did not appear when they talked about the peer situation. The second subtheme concerned responses such as “I do not know” when asked what they would do if they were refused. This was interpreted as indications of children’s inability to predict their actions or see a way to express their agency that would meet with success.

#### Theme 2: child acts with agency

Four subthemes, *emotional resistance*, *negotiation and persuasion*, *attempts to restore balance of power* and *attempts to find a solution*, captured variations in children’s expression of agency. Emotional resistance was expressed nonverbally, verbally or physically. Nonverbal resistance consisted of children’s internal experiences of emotion such as being “sad”, “angry”, “very upset”, or “disappointed” when they failed to influence the other person in an interaction. Verbal resistance such as telling their parents to stop or let them be was only reported in the context of parent–child relationships. Children also reported that they would scream if their act of agency was not respected or paid attention to. Children who said they would physically resist described strategies such as hitting, struggling, or “waving [their] arms”. Physical resistance was expressed only in the situation with parents.

*Negotiation and persuasion* were most often reported in the context of teacher–pupil relationships. Children said that they would use explanations for their request or try to persuade the teacher to reconsider. The children said that they would “try to ask again”, and explain why they do not want to do what the teacher says. For example, children said that they “can get sick in the rain”, they “have no extra clothes”, or “it is not fair”.

The subtheme *attempts to restore balance of power* was used to describe children’s reports that they attempted to assert their own power when they perceived a power imbalance between the adults and children in relationships with parents and teachers. Children said they would “ignore the teacher” or “refuse” to do as he or she said. Children also reported they would “tell the head of the school” presumably to counter the teacher’s power by enlisting the support of an even more powerful authority. In the parent–child relationship context children reported they would address the power imbalance using uncooperative tactics such as “let the parent wait” or “I would take my time”.

The theme of restoring power imbalance was not found in the peer relationship context which presumably was inherently more equal in power. Instead, children reported that they would try to find a mutually satisfying solution, consider the peer’s wishes, or by trying to find other democratic ways of solving the problem. For example, children said they would “discuss the situation”, “take turns”, identify “something that both want”, “ask what a friend wants”, or “mix both games”.

## Discussion

Overall the thematic analyses indicated that there are differences between relationship contexts and that children have a more horizontal relationship with their own age peers than adults, like parents or teachers. Sweden is described as a country where children are considered as equals to adults both in the family (Carlson & Earls, 2001), and in school (Lgr11, 2011). Despite this, our analyses of children’s perceptions of responses to their agency revealed that children felt that their agency was less supported in relationships with the parent and teacher adults in their than in relationships with peers. Children’s responses were in the peer relationship influenced by democratic values, in the parent relationship by closeness and intimacy, and in the teacher relationship by power asymmetry.

When interacting with teachers, children primarily mention negotiation or persuasion as a solution as actions of agency. Also, children’s relationships with teachers was the only context where the children did not think that their agency would be rewarded. Instead children believed that the only context out of these three where their attempts to display agency would be reprimanded was with teachers. One explanation of these results can be that there is more horizontal power in the parent–child and peer relationship (Laursen & Bukowski, 1997), than in a non-close or institutional teacher–pupil relationship (Mayall, 1994). Although the intention in Swedish schools is to encourage children to take more responsibility and to get involved, it is apparent that the inherent role of teachers as institutional authorities may to some extent place limits on children’s perceptions of equality. Past research shows similar results where the majority of schools are organized with class or student councils (Skolverket, 2001), but the students nonetheless feel that they have little or no influence. The finding that children’s perception of their own agency in teacher relations is lacking could also be a result of the competition in the classroom, where many children have to share one, or sometimes two, teachers’ attention. In school, there are more individuals to consider, and teachers work towards a democratic context where there must be consideration of others, in contrast to just offering verbal or physical resistance.

In contrast to teacher–pupil relationships, parent–child relationships are long-term relationships and are more used to shifts in the balance of power than teachers or peers are.

According to Kuczynski and De Mol (2015), power dynamics in parent–child relationships are complex, because the relationship fills many roles including that of providing authority and structure, intimacy and play, as well as caregiving and security. Thus, the nature of power relations, whether it is vertical, or horizontal, whether it is based on authoritative decision making or mutual conflict solving depends on the changing nature of the situations that parents and children encounter. Children are dependent on their parents’ love and support and vice versa. Past research has shown that it is in the parent–child context that children learn argumentation as early as age of three, with continued further progress in arguing (Stein & Albro, 1995). However, in this study, children did not talk about negotiation in a parent–child context at all. Instead, verbal or physical resistance was mentioned in a parent–child context and with peers. Emotional resistance to parents is perhaps not so unexpected since children have a shared history and a closer relation with family members compared to others (Kuczynski & De Mol, 2015). Despite the question in the parent–child context being asked separately for mothers and fathers, none of the children made a differentiation between the parents when they answered. In Sweden, it is in different ways encouraged that both parents in a family take responsibility for bringing up the children. Parents in the same family in Sweden do seem to think alike in their parenting (Sorbring & Gurdal, 2011) and this might be one reason for the children not dividing up their parents in their answers.

As in the teacher and parent contexts children describe situations when their action of agency was unsuccessful. However, in peer relationships they add that next time they might get their own way instead. Child-to-child situations seems to be more democratic, or horizontal in power dynamics where they sometimes “lose” and sometimes “win”. This was not expressed in the same way with adults where power imbalances were more pronounced. Children showed a sense of powerlessness when describing situations with adults, which not was the case with peers. The interaction with the peer is described as the context in which there is the highest possibility to persuade, or just to surrender because maybe next time it will be one’s own turn to decide. Children predict that peer power is equal, or at least changeable, and this makes it possible to take turns deciding from one time to the next. Furthermore, children described the situation with their peers as heterogeneous by saying that it can differ from one situation to the next depending upon which friend is involved in the interaction.

### Limitations and the direction of future research

The large sample of 103 children providing qualitative data offers a view of how Swedish children can think about their agency in different situations. However, because this was not a quantitative study, claims of differences do not mean statistical differences and the result should be taken as suggestive and need to be followed up by future research. The data were presented at group level since the purpose was to find out how children, as a group, believe in their agency in different relationship contexts. Hence, the individual level is not analysed. This means that we have not compared one person’s answers in one context to another context. As a result, we do not know if the same children express low agency in both parent–child and teacher–pupil contexts, or with peers. Thus, future research could explore this comparison. In line with this, it would be interesting to find out to what degree age influences agency in different contexts, with the advantage of following children longitudinally.

Furthermore, the vignettes were constructed to help children picture themselves in different situations, but these situations can be experienced differently, depending upon the child’s background. Although the vignettes might not always be comparable for all children, they do show some variance in the children’s belief in their agency, depending upon the context. Future research should add more vignettes for each context and in that way, collect more data to compare. In this study, we chose one scenario for each context, chosen since most of children experience them in everyday life, but with more vignettes we could have different scenarios.

## Conclusion

This study contributes to the knowledge about how children perceive their agency in different relationship contexts. One conclusion is that children believe they would have agency in all three contexts—with parents, teachers and peers—but that it might be expressed in different ways. Resistance through ignoring or refusing is related to the adult contexts with parents or teachers, while the democratic solution is mainly used with peers. In contrast to this, some children think they would take charge and help out in different ways to solve a problem, although this is shown mainly in the parent context and partly in the peer context. If the result, analysed on a group level, is also valid on an individual level, the results showing that children perceive that they do not have as much agency in school or with parents could be alarming as this can have an impact on their well-being or health. One way of using these results is to work with children and their belief of their own agency since studies show that it is possible to increase individuals’ sense of agency (Adler, 2012). According to Kuczynski and De Mol (2015) there is a dynamic that exists between individuals that can explain the shift in children’s sense of agency. Although parents and children are equally human agents they are unequal in power. When parents and children interact there can be equality between them in their capacities as interpretive, strategic agents but there is still an inequality in terms of the resources that they can draw on when acting as agents. Further, agency in different contexts can differ since individuals are able to adjust depending on with whom they interact (Kuczynski & De Mol, 2015). Indeed, even if a child does not believe he or she can show agency in teacher–pupil relation context, he or she might feel agentic at home. If sense of agency is related to the dynamic between individuals it would also be possible to try to change this or at least try to influence it. Another consideration is that children do use earlier experiences to evaluate and interpret new situations. Results from this study indicate that it could be possible to make even clearer to parents and teachers that they should encourage children to take part in decision-making and give them attention. Encouraging children in this way allows for more experiences where children feel involved and more of a participant instead of just an audience. This can, for example, be done through parenting education, through laws, and in school settings. Developing children’s sense of agency, in parent–child and in teacher–pupil context, can contribute to greater health and well-being in children and youth.

## References

[CIT0001] Adler J. M. (2012). Living into the story: Agency and coherence in a longitudinal study of narrative identity development and mental health over the course of psychotherapy. *Journal of Personality and Social Psychology*, 102(2), 367–9. 21910554 10.1037/a0025289

[CIT0002] Bandura A. (2001). Social cognitive theory: An agentic perspective. *Annual Review of Psychology*, 52, 1–26. 10.1146/annurev.psych.52.1.1 11148297

[CIT0003] Björnberg U. (2002). Ideology and choice between work and care: Swedish family policy for working parents. *Critical Social Policy*, 22(1), 33–52.

[CIT0004] Braun V., & Clarke V. (2006). Using thematic analysis in psychology. *Qualitative Research in Psychology*, 3(2), 77–101.

[CIT0005] Carlson M., & Earls F. (2001). The child as citizen: Implications for the science and practice of child development. *International Society for the Study of Behavioral Development Newsletter*, 2(38), 12–16.

[CIT0006] Côté J. E. (1997). An empirical test of the identity capital model. *Journal of Adolescence*, 20, 577–597. 9368134 10.1006/jado.1997.0111

[CIT0007] Daniels L. M., Perry R. P., Stupnisky R. H., Stewart T. L., Newall N. E. G., & Clifton R. A. (2014). The longitudinal effects of achievement goals and perceived control on university student achievement. *European Journal of Psychology of Education*, 29(2), 175–194.

[CIT0008] Dignath C., Buettner G., & Langfeldt H.-P. (2008). How can primary school students learn self-regulated learning strategies most effectively? A meta-analysis on self-regulation training programmes. *Educational Research Review*, 3(2), 101–129.

[CIT0009] Greenaway K. H., Haslam S. A., Cruwys T., Branscombe N. R., Ysseldyk R., & Heldreth C. (2015). “We” to “Me”: Group identification enhances perceived personal control with consequences for health and well-being. *Journal of Personality and Social Psychology*, 53–74. 10.1037/pspi0000019 25938701

[CIT0010] Grob A., Little T. D., Wanner B., Wearing A. J.,& EURONET (1996). Adolescents’ well being and perceived control across 14 sociocultural contexts. *Journal of Personality and Social Psychology*, 71(4), 785–795. 8888603 10.1037/0022-3514.71.4.785

[CIT0011] Gurdal S., Lansford J., & Sorbring E. (2015). Parental perceptions of children’s agency: Parental warmth, school achievement and adjustment. *Early Child Development and Care*, 186(8), 1203–1211. 27570362 10.1080/03004430.2015.1083559 PMC4999070

[CIT0012] Hallden G. (1991). The child as project and the child as being: parents' ideas as frames of reference. *Children & Society*, 5(4), 334–346. doi:10.1111/j.1099-0860.1991.tb00499.x

[CIT0013] Harach L. D., & Kuczynski L. J. (2005). Construction and maintenance of parent-child relationships: Bidirectional contributions from the perspective of parents. *Infant and Child Development*, 14, 327–343.

[CIT0014] Harcourt D., & Hägglund S. (2013). Turning the UNCRC upside down: A bottom-up perspective on children’s rights. *International Journal of Early Years Education*, 21(4), 286–299.

[CIT0015] James A., & James A. L. (2004). *Constructing childhood: Theory, policy, and social practice*. Basingstoke: Palgrave Macmillan.

[CIT0016] Juang L. P., & Silbereisen R. K. (2002). The relationship between adolescent academic capability beliefs, parenting and school grades. *Journal of Adolescence*, 25(1), 3–18. 12009746 10.1006/jado.2001.0445

[CIT0017] Jutengren G. (2004). Dealing with Integrational Disagreements. *Parental authority in Swedish families* (Doctoral thesis) Department of Psychology. Gothenburg: Gothenburg University.

[CIT0018] Kuczynski L. (ed). (2003). *Handbook of dynamics in parent-child relations*. London: SAGE.

[CIT0019] Kuczynski L., & De Mol J. (2015). Dialectical models of socialization In Overton W. F. & Molenaar P. C. M. (Eds.), *Theory and method. Volume 1 of the handbook of child psychology and developmental science* (7th ed.) (pp. 323–368). Editor-in-Chief: Richard M. Lerner Hoboken, NJ: Wiley.

[CIT0020] Laursen B., & Bukowski W. A. (1997). A developmental guide to the organization of close relationships. *International Journal of Behavioral Development*, 21, 747–770. 20090927 10.1080/016502597384659 PMC2808033

[CIT0021] *Lgr11, Läroplan för grundskolan, förskoleklassen och* fritidshemmet (2011). *[Curriculum for elementary school, preschool class and the leisure center 2011]*. Stockholm: Fritzes (2011).

[CIT0022] Löfdahl A., & Hägglund S. (2006). Power and participation: Social representations among children in pre-school. *Social Psychology of Education*, 9(2), 179–194.

[CIT0023] Lopez D. F., & Little T. D. (1996). Children’s action-control beliefs and emotional regulation in the social domain. *Developmental Psychology*, 32(2), 299–312.

[CIT0024] Markström A., & Hallden G. (2009). Children’s strategies for agency in preschool. *Children & Society*, 23(2), 112–122.

[CIT0025] Matthews S. H. (2007). A window on the ‘new’ sociology of childhood. *Sociology Compass*, 1(1), 322–334.

[CIT0026] Mayall B. (Ed.). (1994). *Children’s childhoods: Observed and experienced*. London: Falmer Press.

[CIT0027] Musher-Eizenman D., Nesselroade J. R., & Schmitz B. (2002). Perceived control and academic performance: A comparison of high- and low-performing children on within-person change patterns. *International Journal of Behavioral Development*, 26(6), 540–547.

[CIT0028] Persson S., Stattin H., & Kerr M. (2004). Adolescents’ conceptions of family democracy: Does their own behaviour play a role? *European Journal of Developmental Psychology*, 1(4), 317–330.

[CIT0029] Russell A, Pettit G. S, & Mize J (1998). Horizontal qualities in parent–child relationships: parallels with and possible consequences for children's peer relationships. *Developmental Review*, (3) (3) 18, 313. doi:10.1006/drev.1997.0466

[CIT0030] Skolverket (2001). *Ung i demokratin. [Youth in democracy] Rapport 210*. Stockholm: Liber.

[CIT0031] Sorbring E. (2005). *Girls´ and Boys´ Views of Conflicts with parents* (Doctoral thesis), Department of Psychology. Gothenburg: Gothenburg University.

[CIT0032] Sorbring E., & Gurdal S. (2011). Attributions and attitudes of mothers and fathers in Sweden. *Parenting: Science and Practice*, 11(2–3), 177–189. 10.1080/15295192.2011.585565 PMC3173942 21927589

[CIT0033] Stattin H., Persson S., Burk W. J., & Kerr M. (2011). Adolescents’ perceptions of the democratic functioning in their families. *European Psychologist*, 16(1), 32–42.

[CIT0034] Stein N. L., & Albro E. R. (1995). Building complexity and coherence: Children’s use of goal-structured knowledge in telling good stories In Bamberg M. (Ed.), *Narrative development: Five approaches* (pp. 5–70). Hillsdale, NJ: LEA.

[CIT0035] Stetsenko A., Little T. D., Oettingen G., & Baltes P. B. (1995). Agency, control, and means and beliefs about school performance in Moscow children: How similar are they to beliefs of western children? *Developmental Psychology*, 31(2), 285–299.

[CIT0036] Vallacher R. R., & Wegner D. M. (1989). Levels of personal agency: Individual variation in action identification. *Journal of Personality and Social Psychology*, 57(4), 660–671. 10.1037//0022-3514.56.2.199 2926623

[CIT0037] Walls T. A., & Little T. D. (2005). Relations among personal agency, motivation, and school adjustment in early adolescence. *Journal of Educational Psychology*, 97(1), 23–31.

